# Body maps of counter-hegemonic midwifery practices in Chile: a collective case study

**DOI:** 10.3389/fgwh.2026.1832260

**Published:** 2026-07-29

**Authors:** Pía Rodríguez-Garrido, Yennifer Márquez-Mosquera, Nadia Salazar-Toro, Pía Villarroel-Vergara

**Affiliations:** 1 Escuela de Obstetricia y Puericultura, Facultad de Salud y Ciencias Sociales, Universidad de Las Americas, Santiago, Chile; 2 Women, Health and Ethics Study Group, University of Barcelona, Barcelona, Spain; 3 Laboratório de Estudos Sociais Sobre o Nascimento, ISCTE-Instituto Universitario de Lisboa, Lisbon, Portugal; 4 Escuela Renacer, Independent Educational Space, Santiago, Chile; 5 Guild Association Maternas Chile, Santiago, Chile

**Keywords:** body maps, Chile, counter-hegemonic practices, decoloniality, midwifery

## Abstract

**Background:**

Global efforts to improve maternal and newborn outcomes increasingly promote respectful, person-centered, and midwife-led models of care. However, their implementation continues to face structural barriers rooted in biomedical paradigms, professional hierarchies, and institutional forms of violence. In Chile, some midwives have developed counter-hegemonic practices that challenge these barriers while strengthening professional leadership and transformative approaches to care. This study explores how such practices contribute to leadership development and care transformation.

**Method:**

A qualitative collective case study grounded in feminist decolonial epistemologies was conducted. Three Chilean midwives participated in a body mapping process, a participatory and reflexive method used to document professional trajectories, lived experiences, and territorial belonging. Data were analyzed through visual interpretation and collective reflection.

**Results:**

Three interrelated processes were identified: critical reflection on biomedical and institutional norms, including experiences of obstetric violence; recovery and validation of community-based and alternative knowledges; and recognition of territory and lived experience as central to respectful and person-centered care. These processes strengthened professional agency and supported the development of situated and socially accountable models of care.

**Discussion:**

Counter-hegemonic midwifery practices function as professional and political forms of leadership that challenge dominant paradigms while promoting collective learning and transformation within health systems.

**Conclusion:**

The experiences of these Chilean midwives highlight how leadership, professional identity, and transformative care practices can emerge through situated, experiential, and community-based forms of knowledge, contributing to current discussions on midwifery education and leadership.

## 1 Introduction

The trajectory of midwifery in Chile is deeply intertwined with the history of childbirth and its institutionalization. There is no established genealogy of the discipline that identifies its key moments or actors; instead, existing historiography focuses on childbirth's medicalization and the marginalization of midwives. This negative representation reflects unequal power dynamics that have devalued women's and pregnant people's knowledge and wisdom, especially within a precarious and assistentialist public healthcare system (1–3).

According to the International Confederation of Midwives (ICM), midwifery care is grounded in partnership, respect, individualized support, and the promotion of physiological reproductive processes (4). Midwives play a central role in advancing woman-centred and person-centred models of care that recognize the social, emotional, cultural, and political dimensions of reproductive health. These principles are increasingly recognized as essential components of high-quality maternal and newborn care globally and provide an important framework for understanding contemporary transformations in midwifery practice and education (5).

As Sadler notes, Chilean healthcare professionals are trained within sexist and androcentric paradigms that reproduce obstetric violence and hierarchy (6). This is visible in midwifery education, where universities continue to teach practices deemed harmful by international standards, such as movement restriction and routine membrane rupture (7). The emphasis on biomedical knowledge over historical and social awareness may limit opportunities to strengthen professional identity and belonging.

It is therefore essential to build a *herstory* of midwifery: one that centers women as historical subjects and highlights the counter-hegemonic practices of midwives from the Global South, who are critical of their training and aware of their social and territorial roles.

Medical hegemony, according to social anthropologist Eduardo Menéndez, is a paradigm that tends to “exclude or limit the impact and recognition of the importance of economic-political and sociocultural factors in the production and solution of health/illness problems” (8). From this, counter-hegemony is understood as “the creation of a social subject that successfully confronts established power […] and offers a general alternative, both in substantive matters and at the territorial level” (9).

We understand counter-hegemonic midwifery practices as forms of professional practice that challenge the dominance of biomedical, patriarchal, colonial, and hierarchical approaches to reproductive care. Within the Chilean context, these practices emerge as responses to institutional forms of violence, professional subordination, and the exclusion of experiential, community-based, and Indigenous forms of knowledge. Counter-hegemonic midwifery seeks to build alternative ways of caring, learning, and relating that recognize the importance of lived experience, collective knowledge, social justice, and territorial belonging.

Territory is understood not only as a geographical space but also as a social, cultural, political, and relational environment through which identities, experiences, and health practices are produced and negotiated. In this context, counter-hegemonic midwifery practices can also be understood as spaces of learning and leadership formation. Through these practices, midwives develop situated, critical, and relational forms of knowledge that challenge traditional educational models. Thus, these experiences not only contribute to transforming care but also offer relevant insights for strengthening midwifery education and leadership within midwife-led models of care.

In this article, we analyze counter-hegemonic midwifery practices and their impact on the midwifery profession in Chile. Specifically, we examine how these practices are embodied, learned, and enacted throughout the professional trajectories of three Chilean midwives and how they contribute to leadership development, professional identity, and transformative models of care.

## 2 Material and methods

### 2.1 Design

This qualitative study is grounded in decolonial feminist epistemologies, which understand knowledge as situated, embodied, relational, and historically located. From this perspective, knowledge production is not detached from lived experience but emerges through the intersections of body, territory, memory, and social relations (10). Decolonial feminist approaches challenge positivist assumptions of neutrality and objectivity, recognizing the legitimacy of experiential and collective forms of knowledge production that have historically been marginalized within biomedical and academic institutions (10, 11).

This research adopted a collective case study design following Stake's approach, which seeks to understand complex social phenomena through the in-depth exploration of a bounded case within its real-life context (35). The case under study was defined as the collective experience of three Chilean midwives engaged in counter-hegemonic midwifery practices. Rather than focusing on individual biographies as the primary object of inquiry, the study aimed to understand how counter-hegemonic practices are developed, embodied, learned, and enacted throughout professional trajectories.

The unit of analysis was therefore the process through which counter-hegemonic midwifery practices emerge and are sustained across participants' personal and professional experiences. The boundaries of the case were defined by: (a) the experiences of three midwives recognized for their engagement in counter-hegemonic practices within Chile; (b) the body mapping process conducted between 2023 and 2024; and (c) the sociocultural and professional context of Chilean midwifery.

Although this study shares characteristics with participatory and reflexive qualitative approaches, the purpose was not to reconstruct individual life histories but to examine a specific phenomenon—counter-hegemonic midwifery practices—and their contribution to professional identity, leadership development, and transformative models of care. Following Stake's typology, this study can be understood as an instrumental collective case study. The interest lies not in the participants themselves but in the phenomenon they illuminate: the emergence and development of counter-hegemonic midwifery practices in Chile. The three participants were selected because their professional trajectories offer particularly rich examples of this phenomenon and allow an in-depth exploration of how these practices are constructed, sustained, and transformed over time.

### 2.2 Study context

Chile is a South American country with 16 regional subdivisions from north to south. One of its main socio-cultural characteristics is a high diversity of Indigenous peoples recognized by Law #19,253 (12). One of the participating midwives identifies as Aymara and reflects on this experience throughout her body map and narrative. It is also important to consider that Indigenous peoples have their own worldviews regarding ways of living, caring, and relating to territory.

This involves a particular way of connecting with the territory, the body, and the processes of health and illness. Supporting these trajectories through midwifery presents a significant challenge for two main reasons: the medical model has eradicated their practices, and there is no intercultural training for health professionals.

### 2.3 Current context of the profession

The midwifery degree programme in Chile is a five-year university-based professional education programme. Graduates obtain the professional title of midwife and are authorized to provide sexual, reproductive, maternal, newborn, and gynecological healthcare. Midwifery is one of the oldest health professions in Chile, with the first formal school established in 1834 at the University of Chile. Currently, there are 33 midwifery programmes distributed across 25 universities throughout the country (13).

To ensure educational quality, universities follow national accreditation standards and competency-based curricula, resulting in relatively homogeneous professional training across institutions. These standards are broadly aligned with international recommendations, including the International Confederation of Midwives (ICM) Essential Competencies for Midwifery Practice (5).

Traditional and Indigenous midwives follow different educational pathways. Their knowledge is generally acquired through intergenerational transmission, apprenticeship, observation, community participation, and experiential learning processes that may extend over many years. Despite their historical contributions to reproductive health, these forms of knowledge remain largely absent from formal university curricula and are frequently marginalized within dominant biomedical frameworks.

### 2.4 Participants' profiles

Four midwives participated in the study process, including the first author and three participant-researchers whose body maps constitute the primary source of analysis. Participants were selected through purposive sampling based on their recognized trajectories in counter-hegemonic midwifery practices, their active involvement in community-based, feminist, intercultural, or alternative models of care, and their willingness to engage in a collaborative and reflexive research process.

Rather than seeking statistical representativeness, participant selection aimed to achieve information power, whereby a smaller sample can generate rich and meaningful data when participants possess extensive experience related to the phenomenon under study. The selected participants represented diverse geographical regions of Chile and different forms of engagement with counter-hegemonic midwifery.

Sample size was determined according to the principle of information power. Given the specificity of the research aim, the depth of participants' experiences, their extensive engagement with counter-hegemonic midwifery practices, and the richness of the visual and narrative materials generated, three body maps provided sufficient information to explore the phenomenon under study. The purpose was not statistical representativeness but the generation of rich and meaningful understandings of the case.

The first author facilitated the research process and participated in all stages of reflexive dialogue and analysis. However, by collective agreement, her body map was not included in the final presentation of results in order to prioritize the experiences of the other participants and to accommodate manuscript length limitations.

### 2.5 Information co-construction technique: body map as a pedagogical and methodological tool

#### 2.5.1 Body map

In this study, body mapping functioned not only as a methodological strategy but also as a pedagogical and reflexive tool. It enabled participants to critically examine their professional trajectories, learning processes, and the development of leadership capacities.

From a decolonial feminist perspective, the body is understood as a site of knowledge, memory, and political agency, allowing the articulation of experiential and situated forms of learning relevant to midwifery education (14).

Using the body map as a knowledge co-construction technique and strategy implies legitimizing the body as a territory which seeks out knowledge, practices, and connections (15). The encystment of colonial, ableist, and patriarchal comprehensions in health (16) has privileged the organic appraisal of the body above the *sentipensante* component (17).

In response to this, from decolonial feminist epistemologies, living, being, and inhabiting the body implies a strong connection and commitment with the territory (10, 11, 18). Everything which happens there affects our bodies and connections, which is why we cannot configure ourselves as people at a distance from our identities, without the pretension of falling into essentialism, but rather highlighting the role which the individual, social, and political body (19) plays across our professional and life trajectories.

##### 2.5.1.1 First stage

The body maps' socialization took place across ten encounters between the four midwives who co-created the article. Considering the geographical distance between us, we supported each other through phone calls, *WhatsApp* messages, and *Zoom* meetings.

The ten encounters lasted between 90 and 120 min and were organized into three phases. The first phase focused on introducing the body mapping process, discussing the study aims, and collectively reflecting on the generative questions. The second phase involved the creation and progressive refinement of the body maps, accompanied by written reflections and narrative accounts. The third phase consisted of sharing, discussing, and collectively interpreting the visual and narrative materials generated throughout the process. Detailed notes were produced after each encounter and incorporated into the analytical process.

In order to respect the extension of the document, three of the four midwives presented their body maps. This was by common agreement with the first author opting to give priority to her colleagues' experiences in order to contribute to other aspects of the study. Although the first author did not include her body map in the final presentation of findings, she participated in all stages of data generation, reflexive dialogue, and analysis. We acknowledge that the absence of her visual narrative may have influenced the analytical emphasis of the study. This decision was collectively discussed and is recognized as a limitation.

##### 2.5.1.2 Second stage

The following generative questions guided the body map co-construction process:

How did my professional trajectory as a midwife take shape?; At what point in my professional/personal life did I start questioning the usual style of the profession?; What counter-hegemonic practices can I identify in my work as a midwife?; How important are the body and the territory in constituting my role as a midwife?; How do I contribute to a midwifery with greater socio-political commitment which can generate a transformative impact on the country?

### 2.6 Ethical considerations

The research was conducted in accordance with the principles of the Declaration of Helsinki. Ethical approval was granted by the Ethics Committee of the University of *anonymised* (IRB: 024–2023).

Given the participatory and reflexive nature of the study, particular attention was paid to ongoing consent, collective decision-making regarding representation, and the management of participants' dual roles as researchers and research participants.

### 2.7 Reflexivity and rigor

Throughout the study, reflexivity was understood as a continuous process of critically examining how personal experiences, professional identities, relationships, and positionalities influenced knowledge production. Three participants occupied dual roles as both research participants and co-authors, while the first author functioned simultaneously as researcher, facilitator, participant, and analyst.

Rather than attempting to eliminate subjectivity, the study embraced positionality as a source of situated knowledge consistent with feminist and decolonial epistemologies. Reflexive practice was sustained through collective discussions, written reflections, field notes, and repeated dialogue across the ten encounters.

Interpretations were initially developed individually and subsequently discussed collectively. Points of convergence and divergence were explored through dialogue until a shared interpretation was reached. This process enhanced analytical transparency, credibility, and confirmability.

To strengthen rigor, the study followed the Standards for Reporting Qualitative Research (SRQR) (20). The use of multiple forms of data (visual representations, oral narratives, and collective discussions), prolonged engagement among participants, and collaborative interpretation contributed to the trustworthiness of the findings.

### 2.8 Data analysis

Image analysis was used, since it provides a situated and decolonial perspective on the way in which we capture reality. In the words of Silvia Rivera Cusicanqui, “Descolonizing perspectives consists of liberating visualization from the bonds of language, and in refreshing the memory of experience as an indissoluble whole, where bodily and mental senses fuse” (21).

The process involved both individual and collective analyses of the body map images, followed by the narration of the experiences they represented. Three key stages structured the analysis: a) Reconstructing memories of our trajectories, by recovering the recollections that shaped our personal and professional histories; b) Projecting bodily geographies, identifying the parts of the body that marked these trajectories; and c) Contextualizing bodily geographies, by incorporating symbols, drawings, and phrases linked to situations, people, and dates that responded to the generative questions.

The body maps were subsequently imbued with meaning through a narrative process carried out first individually and later shared collectively with the midwives who co-created this study. Through repeated readings of the narratives, examination of the visual materials, and collective interpretive discussions, recurring meanings, experiences, and patterns were identified across participants. These recurring elements were progressively grouped into preliminary categories, which were subsequently refined through dialogue among the research team. Analytical decisions were discussed collectively, and divergent interpretations were explored until agreement was reached. This iterative process resulted in the construction of analytical dimensions that captured both the uniqueness of each trajectory and the common elements shared across participants.

Finally, common experiences between all participants were described, arising from coinciding processes in the three narratives, leading them to develop counter-hegemonic practices for Chilean midwifery.

## 3 Results

### 3.1 Central dimension: “woman, mother, and integral family midwife”

Pía identifies as a holistic family midwife. She lives in the central-southern region of Chile and has eighteen years of professional experience working in both public and private hospitals, as an independent practitioner, educator, and home birth attendant. In her body map, she describes four key moments that led her to question traditional midwifery and to engage with the counter-hegemonic practices that shape her current professional approach.

#### 3.1.1 Subdimension: “first awakening (2000–2005)”

**Figure 1:**
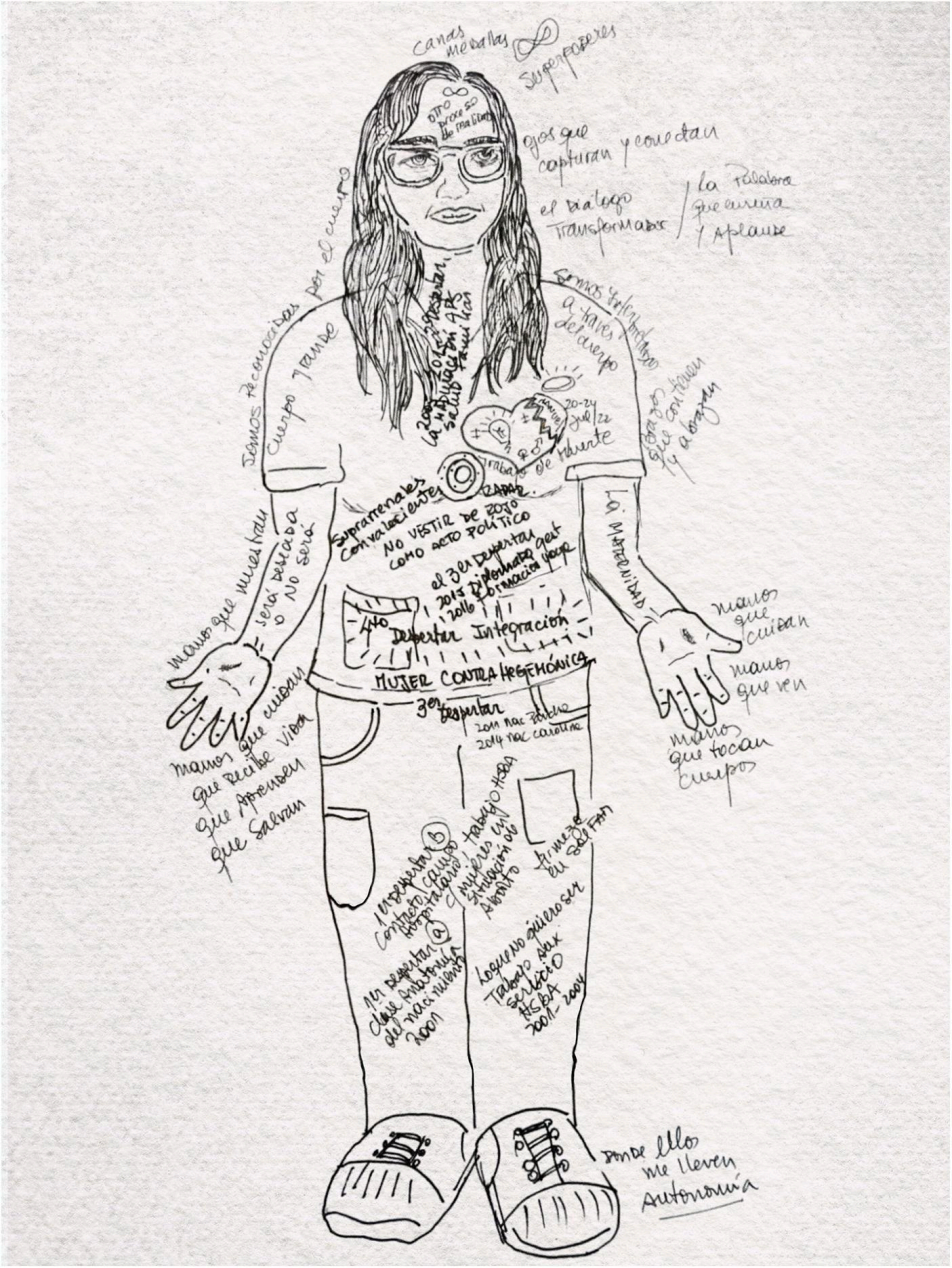
Pía's body map, representing her personal and professional trajectory and the four awakenings that contributed to the development of her counter-hegemonic midwifery practice.

At this stage of her life, which she identifies as her *first awakening*, Pía describes it as part of her formative process; accordingly, she locates it on both legs of her body map. This awakening marked the beginning of her professional deconstruction, as she began to question hegemonic training models rooted in patriarchy and the biomedical paradigm. She also recalls one of her early work experiences in a maternity ward, where she witnessed mistreatment and medical violence against women—an experience that prompted her to reflect on the kind of midwife she did not want to become (see Figure 1 and Table 1).

**Table 1:** Central dimension “woman, mother, and integral family midwife”.

Name	Subdimensions	Body map narratives
Pía	First awakening (2000-2005)	“I place this in the part of the body which lets me walk: the formative process. During this time, I ascribed to different identitarian elements of the profession; while also publicly questioning the care model, the medical patriarchy, and the professional role in every social space where I could. I also worked as a service helper in a maternity ward, which gave me clarity about what I did not want to be, do, or inhabit as a midwife, as well as letting me ‘sentipensar’ other territories for dignified childbirth”.
	Second awakening (2005-2015)	“The transformative word comes from between the heart and the mouth. The main environment for professional maturing has been within primary healthcare institutions. This scenario incorporates community work with families into midwifery praxis; it also creates deep, transformative bonds with vicarious models (Midwives who have been sources of inspiration, support, and motivation) in the form of experienced midwives who created an opening for other health worldviews, developing skills, and deconstructing stereotyped gender beliefs. There was a parallel incursion into the university teaching field, where I once again ‘embraced’ the inclination towards academic life and went into a master’s program which I did not finish. However, in this case I discovered the social sciences, which led me to change my interpretive optics of reality”.
	Third awakening (2011-2016)	“Major life events happened during these years, such as going through diametrically opposed birth experiences between my first son and second daughter. This is also told by my identitarian transit as a woman, mother, midwife, and activist, which is why this awakening is located in the uterus and vagina. The third awakening comes from politicizing motherhood and midwifery, re-embracing the causes of the first awakening and granting them political meaning. In my second postpartum period, I took a diploma course called ‘post-title in gestation, birth, and conscientious labor’ which let me add content and political meaning to my first awakening. Afterwards, I continued training as a pre- and post-natal kundalini yoga instructor, in this case via spiritual and physical work, which deepened the bonds with new vicarious models: midwives for home deliveries”.
	Fourth awakening: from the navel to the center	“Helping with a home childbirth for the first time marks the birth of a continually developing praxis of integrated midwifery which provides organized care for all stages of the individual and family life cycle of women, from birth to death. Placing care at the center of clinical midwifery practice turns out to be obvious, and while care is in the discourse (the guild motto ‘I know how to care for you’), in Chile the profession has an institutional, allopathic, technocratic, and pathogenic orientation. In this context, the permanent construction of a midwifery practice centered on care and wellbeing is a political decision, since this has historically been feminized, invisibilized, and inferiorized. Re-centering the focus on care, from a feminist and intersectional perspective, is thus a revolutionary and counter-hegemonic act, as well as trusting in, using, and valuing our body as a semiological learning and work instrument. In this sense, the body, apart from being the means via which people are recognized and socially interpreted according to the context where we live, is also available as a learning and work instrument which provides a profound connection with midwifery. The stomach which warns when something can go wrong, the hands which can see, touch, relieve, save, and care; the eyes which connect, speak, and capture; the listening which gathers and analyzes; the voice which accompanies, the word which names, contains, or transforms”.

#### 3.1.2 Subdimension: “second awakening (2005–2015)”

Pía situates her *second awakening* between her heart and her mouth. At this stage of her life, she places great value on community-oriented, close-knit, and family-centered work within primary health care, as well as on the learning acquired from experienced midwives. She also highlights the positive aspects of returning to academic teaching. Altogether, these experiences have expanded Pía's professional practice and transformed the way she understands and enacts midwifery (see Figure 1 and Table 1).

#### 3.1.3 Subdimension: “third awakening (2011–2016)”

This awakening is situated between the uterus and the vagina, marking a significant personal stage in Pía's life. Her own experiences of pregnancy, childbirth, and the postpartum period were pivotal in her political awakening as a midwife. Together with her postgraduate diploma studies, these experiences enabled her to explore more deeply the spiritual, physical, and political dimensions of midwifery and home birth (see Figure 1 and Table 1).

#### 3.1.4 Subdimension: “fourth awakening: from the navel to the centre”

In her final awakening, situated at the center of her body, Pía draws on her extensive professional experience to emphasize the need to place women's care and well-being at the heart of the profession, from a feminist and intersectional perspective. This entails not only *being with* people but also respecting their processes, knowledge, and lived experiences. Finally, she highlights the importance of the body as both a site of learning and a tool for practice in midwifery (see Figure 1 and Table 1).

### 3.2 Central dimension: “transformation and rebirth of midwifery”

Yennifer identifies as an independent midwife as a way to reclaim professional autonomy. She lives in the southern region of Chile and has seventeen years of experience working in public hospitals, as an independent practitioner, educator, and home birth attendant. Yennifer highlights five key stages in her personal and professional life as fundamental to the construction of counter-hegemonic midwifery practices, enabling their transformation and renewal.

#### 3.2.1 Subdimension: “professional training: From the intellectual to the social”

**Figure 2:**
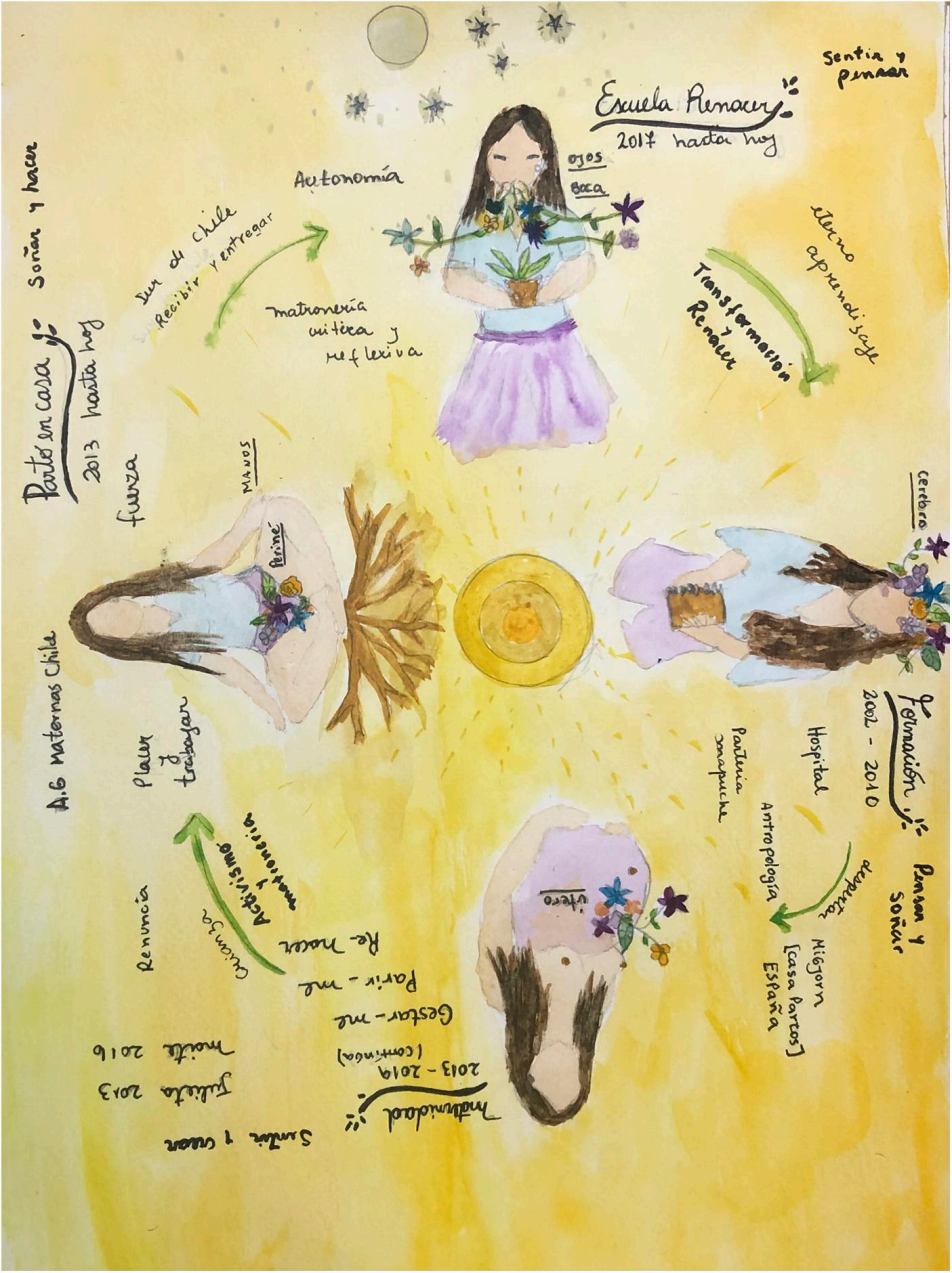
Yennifer's body map, representing the personal, professional, educational, and activist experiences that contributed to the transformation and rebirth of her midwifery practice.

Yennifer situates this moment in her life at the brain of her body map. She emphasizes the importance of skills such as her love of reading and her ability to navigate hierarchies, which have allowed her to cultivate more harmonious relationships within the hospital setting. During this stage, a more critical perspective on professional practice gradually begins to emerge (see Figure 2 and Table 2).

**Table 2:** Central dimension “transformation and rebirth of midwifery”.

Name	Subdimensions	Body map narratives
Yennifer	Professional training: From the intellectual to the social	“During my years of schooling, my attachment to reading (located in the brain of my body map), the search for information, and the closeness with the people who accompanied me in public hospitals helped me sustain a career which at that time was excessively medicalized and violent, although I was not yet aware of it. My abilities helped me deal with hierarchies, apart from learning to work in a hospital system and question its care model, while also valuing the learning and work of people within its system”.
	Approaches to other disciplines: Anthropology as the start of the road	“Since my school days I was close to anthropology, which led me to know about the work of Mapuche midwives, their worldview, and to study their perceptions about childbirth. I did my undergraduate thesis working in Rukas (traditional Mapuche huts) in the urban areas of Santiago de Chile, which drove my interest to learn about anthropology that years later led me to do a Master’s in Anthropology. This also drove me to travel and see the reality of childbirth in other continents. Seeing birthing houses and home births opened up a new door of understanding and identity, and I finally felt pleasure in my work for the first time. My days in ‘Migjorn’ (a birthing house in Barcelona, Spain) were like being on vacation, but in a space for learning and work, since it showed me the physiology of birth, the midwives’ calm, and the simplicity of the birthing spaces”.
	My motherhood: When obstetrics goes through my body	“My gestations, the births of my daughters – the first by Caesarean and the second at home– and living through the postpartum period, have definitely been the most transformative experience of my life, as well as for my identity as a midwife. I feel like gestating my daughters’ lives was like doing it to myself. My uterus grew to give my daughters life, but it also expanded to give me life as a woman and a midwife. In this space, ideas, projects, and decisions started to gestate which would mark my career path. The desire to have children and make it compatible with my work life led me to generate new autonomous work spaces. This fortified my work with social activism, which had begun years before, but became consolidated after my first childbirth, showing the importance of raising my voice for myself and for all women”.
	Home childbirth as a personal and collective decision	“After my personal and professional experiences with home childbirth, I felt like this would be not just a way of work, and that with the years it could become a way of life. The Maternas Chile Guild Association was the space which we built, where we bring together our needs, problems, and dreams so that home childbirth with professional assistance could become visible and take root in our country. Being a home midwife brought me closer to joy and pleasure, the perineal zone (vulva, clitoris, vagina) as energy centers of creation and pleasure identify this space in my life. My hands provide warmth and company in labor and in births, as well as the techniques and procedures to do when any obstetric situation merits such action”.
	*Renacer* School as an expansion space: moving towards transformation and rebirth	“Renacer School is a training space for gestation, labor, birth, puerperium, and breastfeeding, where we provide a critical vision of midwifery to complement medical professionals’ training through knowledge and wisdom from modern obstetrics and traditional midwifery. I place this moment of my life in my eyes. I feel like in every childbirth, my gaze compenetrates women, giving me greater connection and moving to intuition. The voice in the mouth is an important part of my body. Today, through teaching other midwives, I try to transmit my experience and contribute to reconstructing their identity. Being an autonomous midwife and having had a counter-hegemonic trajectory brings me closer to the history of the ‘parteras’, the early midwives who were persecuted and burned, but it has also shown me that I am a woman of today, who accompanies women and families where preserving life and the connection with technology is fundamental, so I value the clinical experience of modern obstetrics, the knowledge I got from the university, and the work in health and education institutions. I am a midwife who carries out practices which vary from those established in the hegemonic health model, I believe in the autonomy of women and midwives, and I want to make a contribution to the transformation and rebirth of midwifery in Chile”.

#### 3.2.2 Subdimension: “approaches to other disciplines: anthropology as the start of the road”

The incorporation of anthropology into Yennifer's professional path proved highly enriching. It provided her with tools to engage with midwifery practices grounded in Indigenous knowledge systems, as well as to understand birth experiences in other countries and within different cultural frameworks (see Figure 2 and Table 2).

#### 3.2.3 Subdimension: “my motherhood: when obstetrics goes through my body”

Yennifer describes her own births—the first by cesarean section and the second at home—as pivotal moments in both her life and the construction of her professional identity as a midwife. The need to balance work and motherhood prompted her to explore new paths and to engage more actively in health activism and the movement for dignified childbirth (see Figure 2 and Table 2).

#### 3.2.4 Subdimension: “home childbirth as a personal and collective decision”

Following her home birth experience and her involvement in activism, Yennifer embarked on a path of dedication and advocacy for home birth. This experience brought her joy, fulfillment, and renewed motivation to inspire other colleagues to join in promoting home births attended by professional midwives (see Figure 2 and Table 2).

#### 3.2.5 Subdimension: “renacer school as an expansion space: moving towards transformation and rebirth”

The creation of *Escuela Renacer*—a training space grounded in a critical perspective on childbirth—represents a major milestone in Yennifer's professional journey. Until its establishment, there had been no comparable space in Chile where counter-hegemonic midwifery practices were taught and strengthened. Within this school, technical knowledge is harmoniously interwoven with ancestral knowledge and insights from other disciplines and crafts. This approach entails recognizing and valuing knowledge that emerges from diverse contexts and integrating it with scientific understanding, thereby fostering the development of professionals committed to women's health from feminist and political perspectives (see Figure 2 and Table 2).

### 3.3 Central dimension: “midwife with the soul of all the midwives that have lived in my life”

Nadia identifies as an Aymara midwife from an Andean community of Abya Yala, located in the northern region of Chile. She has eight years of professional experience working with non-governmental organizations, Indigenous midwives, primary health care services, and home birth practices. Her work with Indigenous communities—both in Chile and the Amazon—constitutes a significant contribution to the development of counter-hegemonic midwifery practices in Chile and across Latin America.

#### 3.3.1 Subdimension: “life mission”

**Figure 3:**
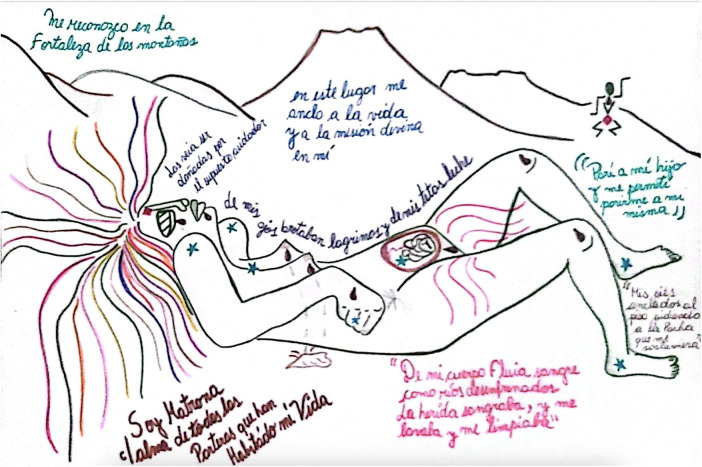
Nadia's body map, representing her personal and professional trajectory, her connection with territory and Indigenous knowledge, and the experiences that shaped her counter-hegemonic midwifery practice.

Nadia recognizes the spiritual dimension of her work as fundamental. The support, care, and companionship she offers to women are deeply meaningful to her, arising from a balance between the human, the spiritual, and the earthly (see Figure 3 and Table 3).

**Table 3:** Central dimension “midwife with the soul of all the midwives who have lived in my life”.

Name	Subdimensions	Body map narratives
Nadia	Life Mission	“A red diamond on my forehead, in the third eye, is what guides my work in this life of accompaniment, service, and containment, the reason why I live in this body. Placed on the hip of a female figure in a yoga posture posed over the mountains, anchoring the spiritual and the human, being the anchor to the earth”.
	*Aymara Warmi:* My indigenous *Aymara* female being	“Symbolized with a turquoise star- From the heart, I place it as a living force, innate and felt from family memory with stories of banishment and forced migrations. Present with the strengthening of my identity in my ayllu^a^ ‘Pachakuti’, here I saw myself and blossom: warmi chamampi^b^. In this space, I grow in knowledge and become an IRPIRI^c^ of my community. I place the responsibility of living as an indigenous person, a daughter of this land, into my shoulders. With a legacy of care for the Pacha Mama^d^ and its people. In my hands is the devotion of recognizing myself and being part of a living people with age-old wisdom. There is also the indication of having knowledge with tremendous empirical wealth, from the search I began 13 years ago with the qollirinaka^e^, usullirinaka^f^, and yatirinaka^g^ women of Abya Yala. This knowledge serves my life in my work as a midwife. My uterus feels the strength of the words from midwives who have nourished my life with absolute goodness, but above all, the ‘tsimana’s midwives in the Bolivian Amazon, where I work with the NGO ‘Bolivia Medical Solidarity’. They are like my sisters and mothers. My steps are strengthened in my feet to walk with strength along the paths of the awichanaka^h^, the heirs of an age-old tradition. There is also my living, present relation with the Pacha ‘My feet anchored to the floor, asking the Pacha Mama to uphold me’”.
	Embodying motherhood in the territories	“An earth-colored drop upon the mountains. From the peaks of the Andes, ‘I recognize myself in the mountains’ strength’. The profound feelings for the place where I live. ‘In this place, I am anchored to life and the divine mission within me’. Upon the eyes and breasts, a phrase emerges ‘tears flowed from my eyes, and milk from my tits’. This is from the horrible experience I had as a patient in a maternity ward with the miscarriage of a very loved and wanted child. The mouth symbolizes when I can speak assertively, educating through feminine spaces and individual care ‘educating in self-knowledge, self-care, and self-healing’ generating bodily empowerment. My fist is raised in a revolutionary sign for the defense of the rights of women, indigenous people, and sexual and reproductive health. This strength is gained by my recognition. There are postgraduate and self-led studies, along with work as a support professional in cultural pertinence for health consultations with indigenous people. The sentence ‘blood flowed from my body like rushing rivers’ emerges from my uterus. The wound (physical and emotional) bled and washed me and cleaned me. After my gestation and childbirth in a miscarriage (which I mentioned before) done in a health service where I suffered obstetric violence, I started to rethink the hegemonic medical model and midwives’ role, and ask: what do I want to offer when giving care? My knees appear in a sign of humility before ancestral midwives’ tremendous knowledge. This spreads in me when carrying out workshops aimed at ‘tsimanes’ midwives to reduce morbidity and mortality among new mothers and babies. This let me understand the role which midwives and their ancestral knowledge play in communities, as ‘guardians of life’. These life experiences, recognizing needs from my indigenous female being, and sharing this feeling from the encounter and bond with my sisters in the towns, let me reflect that the health care process, rather than helping to heal, was imposed and even violent for indigenous people. In this life trajectory, I identify myself as ‘Midwife with the soul of all midwives who have lived in my life’”.

a Community.

b Strong *Aymara* women.

c Authority, respected person.

d Dimension of encounter for space and time with the mother.

e Wise medicine women.

f Midwives.

g Soul doctors.

h *Aymara* grandmothers.

#### 3.3.2 Subdimension: “Aymara warmi: my indigenous Aymara female being”

The stories of exile and forced migration experienced by her community and ancestors provide Nadia with the strength to resist with deeper conviction and a stronger sense of identity. Her journeys across territories and the transmission of knowledge within her community have equipped her with the skills to practice a form of midwifery deeply committed to women's lives. She places particular value on the teachings of women and midwives from other communities and territories (see Figure 3 and Table 3).

#### 3.3.3 Subdimension: “embodying motherhood in the territories”

Territory holds special significance in Nadia's life; she conceives of it as an extension of her own body—a relationship that decolonial feminism describes as *body-territory*. Several profound experiences are inscribed in her body map, including the painful experience of miscarriage and the obstetric violence to which she was subjected. These experiences led her to become an activist for the defense and promotion of women's and Indigenous peoples' sexual and reproductive rights, as well as a facilitator of educational workshops and processes of self-training. All these practices constitute counter-hegemonic expressions of midwifery, as they challenge biomedical hegemony and disrupt the professional status quo (see Figure 3 and Table 3).

The body maps and narratives reveal diverse counter-hegemonic ways of practicing midwifery. Among these practices, we can highlight the questioning of the hegemonic training model inherited from biomedicine; the personal experiences of midwives during pregnancy and childbirth; the respect for individuals' processes, experiences, and knowledge in healthcare; the continuous search for learning from alternative perspectives and disciplines; the harmonious integration of ancestral and scientific knowledge; the activism and politicization of midwifery; and the sustained engagement with communities and territories.

These findings also reveal how counter-hegemonic practices function as spaces for leadership development and situated learning. Through their trajectories, the midwives construct knowledge beyond formal training, integrating embodied, experiential, and community-based perspectives. This contributes to strengthening midwife-led models of care and highlights the transformative potential of alternative approaches to midwifery education.

## 4 Discussion

Critical reflections on the practice and scope of midwifery have gained increasing prominence in recent years. Although most published literature on critical midwifery originates from Europe and the Global North (22–25), similar concerns have emerged in Latin American contexts through decolonial and feminist approaches to reproductive health (2, 3, 14).

Among the most influential contributions within the anglophone literature are Hannah Dahlen's work on the philosophy of childbirth and her critique of obstetric practices (23), Sheila Kitzinger's activism in defense of physiological birth and the politicization of midwifery (24), Ank de Jonge's advocacy for home birth (25), and Ina May Gaskin's efforts to humanize childbirth (26). More recently, the *Critical Midwifery Collective Writing Group* has developed *Critical Midwifery Studies*, an initiative that seeks to confront systemic injustice in sexual, reproductive, maternal, and neonatal care (22).

Although the geopolitical contexts differ, these reflections resonate with the experiences of the midwives in this study. The counter-hegemonic practices of Chilean midwifery intersect with the critical perspectives of European and Anglo-Saxon midwives, particularly in their shared questioning of obstetric practices and the relationships between healthcare professionals, women, and their families.

Beyond their clinical and political dimensions, these findings highlight the relevance of counter-hegemonic practices as sites of learning and leadership development. An important contribution of this study concerns the understanding of leadership as an embodied, relational, and situated process. Rather than emerging from formal managerial positions, leadership developed through critical reflection, community engagement, activism, knowledge exchange, and the capacity to challenge dominant paradigms of care. In this sense, leadership was expressed through everyday practices, educational initiatives, advocacy work, and the creation of alternative spaces for learning and care. This broader understanding aligns with contemporary perspectives that conceptualize leadership as a collective and transformative practice capable of reshaping professional cultures and health systems (5). Furthermore, participants' trajectories demonstrate how knowledge can be generated beyond formal educational curricula through experiential, embodied, and community-based learning processes. These findings suggest the importance of incorporating pedagogical approaches that recognize territory, lived experience, reflexivity, and collective knowledge production as central components of midwifery education.

This perspective resonates with previous research. As noted by the first author and Rojas-Rojas (27) in their study on Chilean midwives' experiences with obstetric violence, ongoing self-critique is essential in healthcare, both individually and collectively. This process must interrogate clinical practices and their implications for relationships with women and their families. Similarly, Gueneau, Larraín, and Schliak (28) problematize the role of Chilean midwives' care throughout history, which, as they argue, “took on a double socio-political function: accompaniment and surveillance” (28). This negative representation has contributed to increasingly distant relationships between women and midwives, especially in institutional contexts. The findings of the present study therefore highlight the complex processes underpinning the participants' trajectories toward counter-hegemonic practices in Chilean midwifery.

One of the key points that emerged from the body maps and narratives was collective learning from others—people, professions, and contexts. The midwives valued the presence of colleagues and non-obstetric learning spaces as essential elements in developing counter-hegemonic practices. A similar perspective appears in Lauren Hunter's (29) study, which explores alternative modes of learning among midwives who attend births. Hunter emphasizes the importance of self-knowledge and experiential understanding of childbirth as fundamental for accompanying birthing people (29). Likewise, Annette Keating and Valerie Fleming (30) highlight intuition, experience, and observation as key sources of midwifery knowledge, paralleling the ways in which the midwives in this study learn beyond “official” or institutionally sanctioned frameworks.

Another common theme across the body maps and narratives was motherhood and mothering as transformative life events. The midwives in this study agreed that their experiences of motherhood provided them with new ways of understanding life and forms of knowledge accessible only through mothering. Sarah Church (31) also observes that midwives, as experiential experts, develop different understandings of risk and safety based on their own birth experiences. However, she cautions that such experiential knowledge can also generate tension between their professional roles and their experiences as mothers.

Similar insights are presented by Coulton–Stoliar, Dahlen, and Sheehan (32), who note that although the knowledge derived from maternity can be empowering, it may also evoke fear and anxiety during pregnancy. This was particularly evident in one participant's narrative and body map, where her experience of obstetric violence during pregnancy became a pivotal moment, motivating her to help other women avoid such harm. This underscores the importance of learning from lived experience to transform obstetric practices.

A third commonality among the narratives and body maps was activism and the politicization of midwifery and motherhood (33). This emerged as a central dimension in shaping the participants' professional identities and counter-hegemonic practices. Jessica Shaw, in *The Medicalization of Birth and Midwifery as Resistance* (34), describes midwifery's position of resistance against the biomedical and interventionist framing of childbirth. Similarly, the chapter *Possible Futures in Complex Times: Epistemic Approaches to a Feminist Midwifery* (3) expands on this idea, emphasizing the political and activist roles of midwives in generating socially transformative change within their territories.

### 4.1 Strengths and limitations of the study

This study's main strength lies in the use of body mapping as a participatory and reflective method that foregrounds the voices, experiences, and embodied knowledge of midwives. The combination of visual and narrative approaches allowed for a deeper understanding of how counter-hegemonic practices emerge, transform, and take root within specific social and territorial contexts. However, as a qualitative and exploratory study with a small number of participants, its findings are not intended to be generalizable. Future research could expand this work by including midwives from other regions of Chile and Latin America, as well as by exploring the reception and impact of counter-hegemonic practices within institutional health settings.

Additionally, the decision not to include the first author's body map in the final presentation may have influenced the analytical emphasis of the findings.

## 5 Conclusion

The findings of this study suggest that counter-hegemonic practices invite midwives to confront contemporary social and cultural challenges through listening, understanding, and empathy. Exploring the personal and professional trajectories of Chilean midwives engaged in these practices reveals both the needs and the opportunities within the profession to strengthen midwifery and to move toward a practice centered on people and their lived experiences—one grounded in deeper political and social commitment.

In this study, three midwives created body maps and narratives reflecting on their professional development and their transitions toward counter-hegemonic midwifery. Three central dimensions and twelve subdimensions were identified, illuminating key ways in which midwives are redefining their professional practice. These include the questioning of the hegemonic biomedical model, the continuous pursuit of learning from diverse perspectives and disciplines, the activism and politicization of midwifery, and sustained engagement with communities and territories. Together, these practices exemplify a form of midwifery that seeks transformation through care, solidarity, and the recognition of embodied and collective knowledge.

Based on the findings of this study, future lines of research could focus on social and cultural midwifery, aimed at understanding how situated and context-specific practices contribute to reproductive justice, intercultural health, and the development of public policies that recognize the diversity of bodies, territories, and the lived experiences of women and birthing people.

These findings also underscore the need to rethink midwifery education, incorporating situated, experiential, and decolonial learning approaches that support the development of leadership capacities. Strengthening these dimensions is essential for advancing midwife-led models of care that are respectful, person-centered, and aligned with human rights.

## Data Availability

The original contributions presented in the study are included in the article. Further inquiries can be directed to the corresponding author.
